# Idiopathic intracranial hypertension: the association between weight loss and the requirement for systemic treatment

**DOI:** 10.1186/1471-2415-7-15

**Published:** 2007-09-21

**Authors:** Roger Wong, Stephen A Madill, Pravin Pandey, Paul Riordan-Eva

**Affiliations:** 1 Department of Ophthalmology, King's College Hospital, London, SE5 9RS, UK; 2 Birmingham + Midland Eye Centre, Dudley Road, Birmingham, B18 7QH, UK

## Abstract

**Background:**

To determine whether weight loss is significantly associated with a discontinuation of treatment for idiopathic intracranial hypertension

**Methods:**

The notes of 36 patients with idiopathic intracranial hypertension under regular review for at least 12 months by a single neuro-ophthalmologist were retrospectively reviewed. Weight was recorded at each assessment and weight loss recommended. Treatment was adjusted according to symptoms, visual function including visual fields and optic disc appearance only. Patients were divided according to duration of continuous follow-up, and then sub-divided as to whether they were on or not on treatment at most recent review and whether weight loss had been achieved compared to presentation. Survival analysis was performed to assess the probability of remaining on treatment having lost weight.

**Results:**

Considering the patients as 3 groups, those with at least 12 months follow-up (n = 36), those with at least 18 months follow-up (n = 24) and those with 24 months or more follow-up (n = 19), only the group with 24 months or more follow-up demonstrated a significant association between weight loss and stopping systemic treatment (Fisher's exact test, p = 0.04). Survival analysis demonstrated that the probability of being on treatment at 5 years having gained weight was 0.63 and having lost weight was 0.38 (log rank test, p = 0.04). The results suggest that final absolute body mass index is more important than the change in body mass index for patients who stop treatment (Mann Whitney U, p = 0.05).

**Conclusion:**

This is the first study to demonstrate that weight loss is associated with discontinuation of treatment. Unlike previous studies, our results suggest that final absolute body mass index is more important for stopping treatment than a proportional reduction in weight.

## Background

Idiopathic intracranial hypertension (IIH) is associated with elevated body mass index (BMI) [1,2]. Recent weight gain at presentation and BMI greater than 40 are predictive of greater visual impairment [3-5]. Weight loss, by dieting or gastric surgery, is associated with normalisation of central serous fluid (CSF) pressure and improvement in papilloedema [1,6-9]. Although these are reasonable short-term measures of disease amelioration, long-term benefit has yet to be demonstrated.

Monitoring disease activity in IIH requires assessment of several factors. Although CSF pressure is the primary parameter, intracranial pressure monitoring has shown that CSF pressure fluctuates widely throughout the day in IIH [10] such that lumbar puncture provides only limited information on disease control [3]. Papilloedema is an important secondary measure but optic disc assessment, even by scanning laser ophthalmoscopy, does not necessarily differentiate between resolving papilloedema and secondary optic atrophy [11]. Visual function, especially visual fields, is a vital parameter but is prone to testing variability and generally can only determine whether there is disease progression not resolution. Assessment of symptoms, particularly headaches, is subject to confounding factors such that their persistence does not necessarily correlate with disease activity. In order to circumvent these difficulties, the end point chosen by this study was the decision by a single neuro-ophthalmologist, based on an overall assessment of these various factors (excluding the patient's weight), as to whether continuation of treatment was required. A particular benefit is that the results provide information easily conveyed to patients about the value of weight loss in IIH.

## Methods

Case notes were reviewed of patients with a diagnosis of IIH fulfilling standard diagnostic criteria [12] identified from a database of patients managed by a single neuro-ophthalmologist since February 1998. The minimum required follow-up for inclusion was 12 months. Ethical approval for this study was granted by the King's College Hospital Research Ethics Committee. Research was carried out in compliance with the Helsinki Declaration.

Symptoms, particularly headache, tinnitus and visual disturbance especially transient visual obscurations, visual acuities, visual fields by Humphrey^® ^24-2 or 30-2 computerised or Goldmann perimetry, optic disc appearance and weight were recorded at each clinic visit. Weight measurements were performed by a nurse using manual scales but without a protocol regarding the amount of clothing that the patient should be wearing.

Weight loss was recommended to every patient, aiming for an initial 5% reduction in weight over 3 months. No specific advice was given as to the method of weight loss, other than reduction in calorie intake and increase in exercise. Referral to a dietician was arranged if requested or there was failure to lose weight. Although an influence on treatment decisions cannot be excluded, weight was not used as a factor in determining treatment. It was used as a factor in advising patients on likely prognosis.

Treatment, determined by symptoms, visual function and optic disc appearance, was primarily acetazolamide, usually starting at 500 mg/day rising to 1 g/day and occasionally up to 1.5 g/day. Furosemide 20–40 mg/day or if headaches were troublesome topiramate gradually increasing to 200 mg/day were used if there was intolerance, lack of efficacy or contraindication to acetazolamide. Generally headaches were treated with paracetamol (acetaminophen), aspirin or non-steroidal anti-inflammatory drugs (NSAID). Opiates and analgesics containing caffeine were avoided or discontinued wherever possible. Surgical treatment was undertaken if there was significant risk of progressive visual loss despite medical treatment either due to lack of efficacy, intolerance or non-compliance. Lumbo-peritoneal shunt was the preferred option, unless the cerebellar tonsils were relatively low, in which case ventriculoperitoneal shunt was performed. Optic nerve sheath fenestration was performed if there was no associated headache or the patient declined a CSF shunt.

We applied 2 exclusion criteria for the review: 1 for pregnancy and 1 for previous surgery. Patients who were pregnant at presentation or had a pregnancy recorded in the notes less than 1 year after stopping systemic treatment were excluded since there would be reasonable grounds to suggest that the pregnancy had influenced the treatment course. With regards to surgery, patients having had a procedure for IIH during the time of follow-up that led to a cessation of treatment were excluded. However those who had a procedure to manage IIH many years before presenting to our clinic or those whose systemic treatment continued unchanged before and after their procedure were included.

The study patients were divided according to the length of time between first and last recorded clinic appointment. The data was sequentially analysed as 3 groups: those patients with follow-up data for 24 months or more from presentation, those with follow-up data for 18 months or more from presentation (which would include all the patients in the 24 month group) and those with follow-up data for 12 months or more (which would include all the patients above). Since the groups were summative, the 12 month group was the largest. As discussed in the introduction, there are limitations associated with using both symptomatology and objective clinical findings as indicators of disease stage in IIH. In searching for a more robust measure of overall clinical improvement we therefore divided the patients for each follow-up group simply according to whether they were on treatment or not on treatment at most recent review. Reflecting the practice of a single neuro-ophthalmologist, the patients' weights were consistently not referred to in making decisions regarding treatment. No other patient parameters were considered in the analysis. We did not subdivide the patients according to the specific treatment prescribed but only the absolute treatment status. The patients who had lost weight at their last review compared to presentation were separated from those who had gained weight. The 4 groups for each period of follow-up were cross-tabulated and analysed using Fisher's exact tests for significance. Since the patient's treatment status at their last review was taken as the single measure of disease behaviour, patients who never required treatment were included in the 'not on treatment at most recent review' groups. Survival curves were plotted to determine the probability of stopping treatment having lost weight over time and the significance of the difference between the curves calculated using a log rank test.

## Results

The notes of 70 patients who had presented sequentially to a single consultant's neuro-ophthalmology practice between February 1998 and March 2003 were retrieved. 41 IIH patients were eligible for inclusion with follow-up of 12 months or greater. All patients met the modified Dandy criteria with secondary causes of raised intracranial pressure excluded using appropriate investigations.

3 patients had surgical interventions (all lumbo-peritoneal shunts) leading to systemic treatment being stopped and were excluded. 2 patients were included with a surgical intervention. 1 had optic nerve sheath fenestration in 1992 and presented in 1999 and 1 had optic nerve sheath fenestration during the follow-up period but medical treatment remained unchanged before and after surgery. 2 patients were excluded who became pregnant within 12 months of stopping treatment. 2 patients were included who became pregnant during the follow-up period but the pregnancy was recorded in the notes 18 months or more after systemic treatment was stopped. We therefore assume that the 2 events are unrelated. 36 patients were therefore included in the analysis, 35 female and 1 male. Mean age at presentation was 31.6 yrs (standard deviation = 10.1 yrs) and mean BMI at presentation was 36.6 kg/m^2 ^(standard deviation = 7.5 kg/m^2^). Mean length of follow-up was 30 months (standard deviation = 16.3 months). One patient had weights but not height recorded. She is therefore included in the cross-tabulations but not the statistics that require BMIs.

The cross-tabulations are shown in table 1 with corresponding p-values from Fisher's exact tests. The cross-tabulation for 12 months or more follow-up contains all 36 patients. 24 of these patients had 18 months or more follow-up and 19 of these patients had 24 months or more follow-up, and were cross-tabulated accordingly. There were no patients with identical BMIs at first and last reviews.

**Table 1:** Cross-tabulations for 12 months and above, 18 months and above and 24 months and above follow-up groups with p-values from Fisher's exact tests

**Length of follow-up**	**Patient's BMI change comparing initial to most recent review**	**Patients on treatment at most recent review**	**Patients not on treatment at most recent review**	
12 months and above				
	BMI increase	12	5	
	BMI decrease	9	10	n = 36
		p = 0.14		
				
18 months and above				
	BMI increase	8	4	
	BMI decrease	4	8	n = 24
		p = 0.11		
				
24 months and above				
	BMI increase	8	4	
	BMI decrease	1	6	n = 19
		**p = 0.04**		

There must be no statistically significant difference between the patients leaving the analysis at each time point and those remaining or the results will be skewed. We divided the patients into 3 groups (different from those used for the cross-tabulation): patients leaving the analysis before the 18 month follow-up point (Group A: 12 patients), patients with between 18 and 23 months inclusive follow-up, i.e. leaving between the 18 and 24 month follow-up points (Group B: 5 patients) and patients with 24 months or greater follow-up (Group C: 19 patients). Using Mann-Whitney U-tests, there was no difference in BMI distributions at presentation when comparing Group C to Group A (p = 0.14) or Group B (p = 0.41). Similarly there was no significant difference between the 3 groups at 12 months follow-up (Group A vs Group C: p = 0.09, Group B vs Group C: p = 0.91) and no significant difference between Group B and C at 18 months follow-up (p = 0.73).

We also divided the patients into all those on treatment at their last review (n = 21) to all those not on treatment at last review (n = 16) to ensure that the 2 groups were otherwise comparable. The results are shown in table 2. There is no statistically significant difference between the groups with regard to age, opening pressure, length of follow up or BMI at presentation.

**Table 2:** Comparing all the patients on treatment at most recent review to all the patients not on treatment at most recent review with regards to parameters indicated

	**Patients on treatment at most recent review**	**Patients not on treatment at most recent review**	**Mann Whitney U test**
Age/years (standard deviation)	32.0 (10.6)	31.2 (10.4)	p = 0.82
Sex	21 female	14 female: 1 male	
Opening pressure/cm H_2_O (standard deviation)	35.9 (11.4)	34.2 (10.4)	p = 0.66
Length of follow up/months (standard deviation)	32.1 (15.5)	32.8 (15.5)	p = 0.90
Body mass index at presentation/kg/m^2 ^(standard deviation)	38.0 (8.0)	34.4 (6.4)	p = 0.17

**Figure 1:**
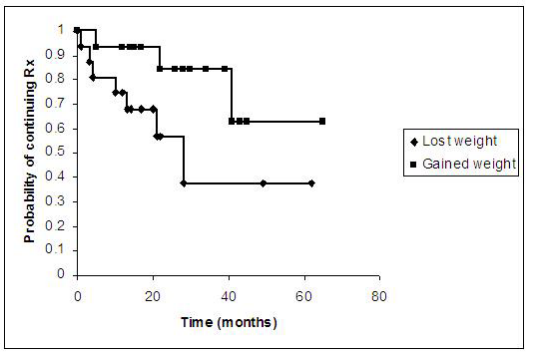
Survival analysis demonstrating the probability of being on treatment having gained weight and the probability of being on treatment having lost weight over time. The end point for the analysis was stopping treatment.

The survival analysis for the patients is demonstrated in figure 1. Since the end point for the analysis was stopping treatment, the patients who were never on treatment or who were followed up for a period before starting treatment were excluded (5 of 36 patients). We divided the remaining 31 patients into gained weight and lost weight groups disregarding the patient's treatment status at last review. In the gained weight group 6 patients had less than 24 months follow-up and 9 patients had 24 months or more follow-up. In the lost weight group 13 patients had less than 24 months follow-up and 3 patients had 24 months or more follow-up. The survival analysis demonstrates that the probability of being on treatment at 5 years follow-up having gained weight is 0.63 whereas the probability having lost weight is 0.38. Significance testing the difference between the 2 curves with a log rank test returns p = 0.04.

We can draw inferences on the amount of weight loss required to stop treatment if we consider only the patients who lost weight (18 of the total 36 patients, excluding the patient with weights but not BMIs: see table 1) and compare those on treatment at the most recent follow-up (9 patients) to those not on treatment at the most recent follow-up (9 patients). The data are presented in table 3. There is no significant difference between those on treatment and those not on treatment with regard to final weight in kilogrammes (Mann Whitney U, p = 0.22) or weight lost in kilogrammes (Mann Whitney U, p = 0.19). Similarly there is no significant difference between the 2 groups when comparing the change in BMIs between first and last reviews (Mann Whitney U, p = 0.26). However the difference between the groups is significant when comparing the final BMIs (Mann Whitney U, p = 0.05). The average final BMI in the group remaining on treatment at last review was 37.6 kg/m^2 ^(95% confidence interval: 30.6 kg/m^2^, 44.7 kg/m^2^) whereas the average final BMI in the group not on treatment at last review was 30.2 kg/m^2 ^(95% confidence interval: 25.2 kg/m^2^, 35.2 kg/m^2^)

**Table 3:** Comparing the final weight, final BMI, change in weight and change in BMI between patients who had lost weight but were still on treatment at their most recent review to patients who had lost weight and were not on treatment at most recent review

	**Patients on treatment at most recent review (95% confidence interval)**	**Patients not on treatment at most recent review (95% confidence interval)**	**Mann Whitney U test**
Number of patients	9	9	
Mean final weight/kg	92.3 (74.9, 109.6)	81.8 (66.2, 97.3)	p = 0.22
Mean change in weight/kg	6.6 (1.2, 12.0)	8.9 (4.5, 13.4)	p = 0.19
Mean final BMI/kg/m^2^	37.6 (30.6, 44.7)	30.2 (25.2, 35.2)	**p = 0.05**
Mean change in BMI/kg/m^2^	2.7 (0.4, 5.0)	3.3 (1.7, 5.0)	p = 0.26

## Discussion and conclusion

Our results demonstrate an association between weight loss and stopping systemic treatment but only after 24 months of follow up. The results suggest that the groups of patients leaving the statistical analysis at each time point are representative of the group as a whole with regard to baseline BMIs and change in BMIs over time. The only significant difference between the groups at each time point from the data collected is therefore length of follow-up and consequently amount of weight lost. The difference between the weight loss and weight gain groups is further supported by survival analysis with a significantly lower probability of being on treatment at 5 years follow-up having lost weight. Unlike previous studies, the results suggest that rather than an average amount of weight loss being associated with improvement, the final absolute BMI is more important for patients stopping treatment.

One previous study comparing weight loss to improvement in objective clinical findings found a significant improvement in papilloedema grade in the patient's worst eye at baseline when comparing the papilloedema grade at the last recorded clinic review of a group of patients who lost 2.5 kg or more over any 3 month interval during follow-up to a group who lost less than 2.5 kg over any 3 month period^1^. The last clinic review was classified as being more than 6 months from presentation in all patients. There was no association between weight loss and improved visual function or visual fields. There was also no correlation between amount of weight loss and degree of papilloedema improvement. Only Johnson *et al.*[9] to date have demonstrated a qualitative association between weight loss and papilloedema resolution reporting that a 6.2% weight loss (standard error of the mean = 0.6%) was significantly associated with a 3 grade improvement in papilloedema using a modified Frisén scale [13] and masked graders. Our study is the first to use the decision to stop treatment taken independently of the patient's weight as a measure of overall clinical improvement. We have attempted to be rigorous in using the patient's treatment status as the sole outcome variable and thus avoid the problems associated with using objective clinical findings or symptomatology to monitor disease remission, as discussed in the introduction. Our study agrees with previous work demonstrating the importance of weight loss. However our results disagree by suggesting that final BMI is more important than the drop in BMI when considering patients who stop treatment.

We must offer a hypothesis for why 24 months of follow-up was required for our weight loss group. We assume that following our weight control protocol takes 24 months for a significant proportion of our patients to lose weight and for treatment then to be stopped. Therefore, although the length of time required will probably be different for different departments, this does not negate the suggestion that weight loss offers continuing benefit with regard to remaining off treatment, with the cumulative effect of steady weight loss manifesting as increasing statistical significance over time for both the Fisher's exact tests and survival analysis. This is not merely an example of prolonged follow-up leading to an inevitable trial discontinuation of treatment or spontaneous disease remission by 24 months for if this was the case then there should be no difference at all between the weight gained and weight lost groups at the 24 month point. We also note however that since this is an uncontrolled study, we are unable to comment further on the natural history of the disease.

Due to problems in the past with monitoring IIH objectively, we suggest that discontinuation of systemic treatment is a robust measure of improvement in the overall clinical condition provided that decisions regarding the patient's treatment are consistently taken independently of their current weight, as in this study. Although the pathophysiological link between body mass and the risk of developing IIH demonstrated epidemiologically remains unclear, we hope that our study contributes useful evidence to the debate regarding the benefit of weight loss for IIH patients.

## Competing interests

The author(s) declare that they have no competing interests.

## Authors' contributions

RW drafted manuscript, gathered data, conducted statistical analysis. SAM conducted statistical analysis and edited manuscript. PP participated in data gathering. PRE conceived the study and participated in design and coordination. All authors read and approved the final manuscript.

## Pre-publication history

The pre-publication history for this paper can be accessed here:


